# On Two Cases with Autosomal Dominant Hyper IgE Syndrome: Importance of Immunological Parameters for Clinical Course and Follow-Up

**DOI:** 10.1155/2020/6694957

**Published:** 2020-12-02

**Authors:** Snezhina Mihailova Kandilarova, Spaska Stoyneva Lesichkova, Nevena Todorova Gesheva, Petya Stefanova Yankova, Nedelcho Hristov Ivanov, Guergana Petrova Stoyanova, Penka Ilieva Perenovska, Marta Petrova Baleva, Elissaveta Jordanova Naumova

**Affiliations:** ^1^Department of Clinical Immunology with Stem Cell Bank, University Hospital “Alexandrovska”, PID National Expert Center, Medical University, Sofia, Bulgaria; ^2^Department of Pediatric Diseases, University Hospital “Alexandrovska”, Medical University, Sofia, Bulgaria

## Abstract

Autosomal dominant hyper-IgE syndrome (AD-HIES) is a rare disease described in 1966. It is characterized by severe dermatitis, a peculiar face, frequent infections, extremely high levels of serum IgE and eosinophilia, all resulting from a defect in the *STAT3* gene. A variety of mutations in the SH2 and DNA-binding domain have been described, and several studies have searched for associations between the severity of the clinical symptoms, laboratory findings, and the type of genetic alteration. We present two children with AD-HIES–a girl with the most common *STAT3* mutation (R382W) and a boy with a rare variant (G617E) in the same gene, previously reported in only one other patient. Herein, we discuss the clinical and immunological findings in our patients, focusing on their importance on disease course and management.

## 1. Introduction

In 1966, Davis et al. [1] published two interesting clinical cases in Lancet: two red-haired white-skinned girls who presented with severe dermatitis several weeks after birth, recurrent cutaneous staphylococcal abscesses, sinusitis, and treatment-resistant pulmonary infections. They named this new nosological entity “Job Syndrome.” None of the parents and siblings shared similar complaints. Six years later, Buckley et al. [2] described two boys with severe dermatitis, a characteristic face, frequent infections, extremely high levels of serum IgE and eosinophilia, and termed the disease “Buckley's syndrome.” In 1974, Hill et al. [3, 4] found that patients with similar diseases had high levels of serum IgE and defects in the chemotactic function of granulocytes. The syndrome was designated as Job's-Buckley syndrome or “Hyperimmunoglobulin E recurrent infection syndrome-HIES.” The disease is autosomal dominant and belongs to the family of primary immune deficiencies (PIDs). In 2004, Renner et al. described six families with different characteristics of hyper IgE syndrome, which was inherited in an autosomal recessive manner [5]. In 2007, genetic defects in the *STAT3* were demonstrated in the autosomal dominant form [6, 7], in 2009 in the *DOCK8* gene [8] and in 2006–2007 in the *TYK2* gene in an autosomal recessive form [9, 10]. Subsequently, several articles reported data on a different number of patients, detecting both established and new mutations [6, 11–15].

In this article, we present 2 children with an autosomal dominant form of HIES (AD-HIES)—a girl with one of the most commonly detected *STAT3* mutations and a boy with a very rare mutation in the same gene. We discuss the clinical and immunological findings in patients and the importance of determining the cytokine profile for disease evaluation.

## 2. Case Presentation

### 2.1. Case 1

**Figure 1:**
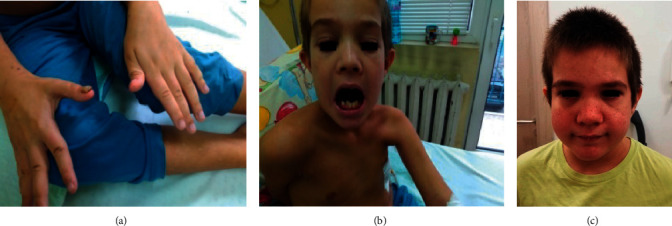
Data of some physical manifestations of Patient 1. (a) Onychomycosis of the thumbnail. (b) Retained deciduous teeth at 7 years. (c) Facial features at 11 years.

Patient 1 is a 10-year-old male who was born full-term [16]. He has no siblings. Family history for PIDs was negative. Vaccines were given on schedule. Since infancy, he suffered from recurrent staphylococcal skin infections, bacterial otitis at 2 months, subcutaneous abscess of the hairy part of the head at 10 month, and styes of both eyelids at age 3. At age 4, he had pneumonia. At age 8, he developed pleuropneumonia complicated by empyema and pulmonary abscesses with multiple pneumatoceles. Imaging studies of the lungs conducted in the past and during the current examination revealed numerous changes such as emphysema, pneumofibrosis with adhesion, atelectasis, pleural effusions, and partial pneumothorax. Physical examination at admission revealed a polymorphic erythematous rash of the face and eyelids, dry skin with hyperpigmentation on the limbs, onychomycosis of the nails (Figure 1(a)), a dolichocephalic configuration of the head, dysmorphic face (Figure 1(c)), and multiple dental abnormalities: retention, hyperdontia, and alignment of the teeth in two rows (Figure 1(b)). Allergy to nuts, house dust, and cow's milk protein has been proven. Microbiological investigation of sputum/throat smear showed various pathogenic microorganisms such as *group A beta-hemolytic streptococci*, *Moraxella nonliquefaciens*, and *Streptococcus pneumoniae*. Bone density was estimated from spinal densitometry and was age-relevant. The definitive diagnosis was made at age 8. Genetic testing showed a heterozygous variant p.1850 G > A (p.Gly617Glu) in exon 20 of the *STAT3* gene, which encodes a transcription factor with key gene regulation activity. According to the criteria of the American College of Medical Genetics, the described variant was categorized as probably pathogenic and, in principal, could explain the observed clinical symptoms. The child was monitored for 4 years after admission. Long-term prophylaxis with Itraconazole 100 mg/day and Sulfamethoxazolum/Trimethoprimum 960 mg daily dose, three times per week, was initiated. A sufficient clinical response was achieved with no severe infections.

### 2.2. Case 2

**Figure 2:**
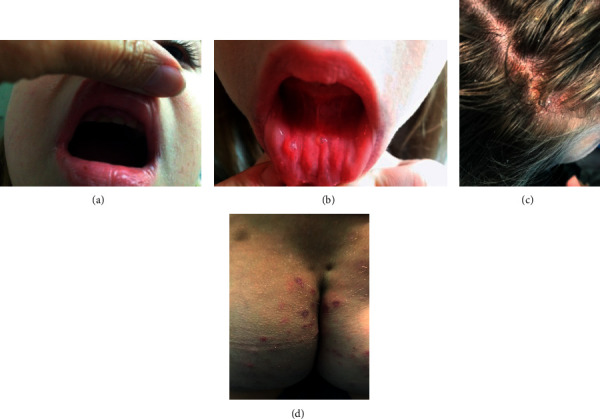
Data of some physical manifestations of Patient 2. (a) Hypoplasia of upper teeth at 7 years. (b) Recurrent oral ulcers at 7 years. (c) Resistant to treatment dermatitis to the scalp. (d) Resistant to treatment dermatitis of the gluteal area.

Patient 2 is an 11-year-old female, born full-term. She has no siblings and no family history for PIDs. The child was vaccinated with no side effects or complications. No dysmorphic features or skeletal abnormalities were noted; however on the physical examination, hypoplasia of the upper teeth and oral ulcers were observed (Figure 2(a) and 2(b)). Since the age of 1 year, she has been having recurrent fungal infections of the oral cavity, skin, and nails *(Candida albicans*, *Zygomycetes species)*. The patient has had several manifestations of bronchial obstruction from infancy. At 18 months, she had pneumonia with pleural empyema and subsequent lobectomy. At age 2, she developed diffuse fibrinopurulent peritonitis with necrosis and colon transversum perforation resolved by surgical intervention. At age 2.8, she was hospitalized with hydrothorax and at age 3, was admitted with peritonsillar abscess. At age 4, she had pneumonia and pleuritis and at age 6, retroperitoneal abscess, subphrenic abscess, and diffuse fibrinopurulent peritonitis. The microbiological investigation at that time revealed *Candida albicans* in feces and throat swab, *Proteus mirabilis* in abdominal exudate, *Klebsiella pneumonia*, *Acinetobacter baumannii*, and *Stephanoascus ciferrii* in a hemoculture specimen. Densitometric studies revealed that bone density was within the expected for her age. The diagnosis was confirmed at age 7 by the presence of the heterozygous R382W germline mutation in the *STAT3* gene. The patient was put on prophylaxis with Itraconazole (10 mg/kg/day) and Sulfamethoxazolum/Trimethoprim at a dose of 480 mg twice per day every other day with relatively good clinical response. Her dermatitis persists despite treatment and prevention (Figure 2(c) and 2(d)). Furthermore, at age 10, hyperplasia of the thymus was observed. However, despite the therapy, in the seventh year of follow-up, there was a worsening of the existing pneumatocele complicated with abscess. *Aspergillus fumigatus* was isolated from sputum.

Hyper IgE Syndrome Scores, according to Grimbacher et al. [17] and *STAT3* variants of both cases, are presented in Table 1. Data from immunological tests are shown in Tables 2–4.

Written informed consent was obtained from the parents of Patient 1 and from the mother of Patient 2.

## 3. Discussion

### 3.1. Increased Serum IgE

In 97% of the patients, IgE levels are above 2000 IU/ml [15]. The diagnostic sensitivity of the elevated IgE levels is 95.8%, but the specificity is very low −3.3% [14]. In the course of the disease, a decrease and even a normalization of high serum IgE levels have been observed in some patients [18]. So far, there is no satisfactory explanation for the cause of the extremely high serum IgE levels in patients with AD-HIES. The following hypothesis has been discussed: association with IL-21 signaling [19, 20], low catabolic rate of IgE [21], unconventional way of binding of *S. aureus* superantigens with MHC class II molecules, the inclusion of a much larger number of T-cell receptors, production of IgE antibodies to staphylococcal superantigens, and massive cytokine production [22–25]. The data concerning food allergy in AD-HIES are controversial: Gernez et al. [26] have found allergy to food in 37% of AD-HIES patients, Siegel et al. [27]–in 8, 5%, Chandesris et al. [15]−8% of AD-HIES patients had asthma and 22%-allergic symptoms mainly food and pollen allergy. Therefore, these reactions are rare in AD-HIES, may possibly be a result of impairment of mastocyte and basophil degranulation, but symptoms of allergy have been described in AD-HIES. This fact points to the importance of diet in these patients, especially in the presence of food allergies. Both patients described by us have elevated levels of IgE (Tables 1 and 2). Patient 1 had a history of rash after oral administration of Amoxicillin/Clavulanic acid, while at the same time allergy to nuts and cow's milk protein has been proven (results not shown). Patient 2 had recurrent pulmonary aspergillosis and showed a trend of increasing IgE levels over the years: from 431 IU/ml at the time of diagnosis to 22 400 IU/ml. In this case, the course of the dermatitis is relatively severe and resistant to treatment. The progressive increase of the disease in Patient 2 might be associated with inadequate infection control and could be a marker of persistent aspergillosis. We suggest that the regular monitoring of IgE titer is important for patients with AD-HIES.

### 3.2. Hypereosinophilia

Hypereosinophilia is due to the increased production of granulocyte-monocyte colony-stimulating factor (GM-CSF) [28, 29]. Eosinophils in the blood are elevated in 70–93% of patients [15], but no correlation was found with IgE levels and clinical symptoms [18]. The diagnostic value of hypereosinophilia in AD-HIES has 93.5% sensitivity, but the specificity is low −23.3% [14]. Mild hypereosinophilia was observed in both patients; however, the higher values in Patient 1 were not related to a worse disease course. The number of eosinophils did not correlate with the measured GM-CSF levels in both cases (Table 3).

### 3.3. Skin Manifestations

The main skin manifestations in AD-HIES are eczema (90%), neonatal rash (45–74%), and skin abscesses (85%) (11, 12, 14, 15). In some cases, the rash is difficult to be distinguished from atopic dermatitis. The typical localization and the presence of lichenified plaques of the anterior neck, antecubital and popliteal fossa in atopic dermatitis come into consideration here. In addition, in AD-HIES, dermatitis is very prolonged, severe, and methicillin-resistant. Involvement of the skin, nails, and mucous membranes in fungal infections is another common manifestation of the disease and is found in 43–85% of patients (11, 12, 14, 15). During infancy, Patient 1 had staphylococcal pyoderma and onychomycosis, but at the same time, he also presented with signs of atopic rash on the flexor surfaces of the limbs and eyelids, drug-induced rash, and allergy to nuts and cow's milk protein. Dermatitis in Patient 2 was persistent, mainly affecting the head and buttocks without a satisfactory therapeutic response. Nails, oral cavity, skin, and intestines were affected by *Candida albicans* and *Zygomycetes spp*.

### 3.4. Pulmonary Manifestations and Severe Infections

Pneumonia and pneumatocele are established in 90–100% and in 45–74.5% of patients, respectively (11, 12, 14, 15). Our subjects were suffering from frequent pneumonia and pneumatoceles formation. A more severe course related to pulmonary complications was observed in the case of the common AD-HIES mutation. Life-threatening infections have been observed in 43–89% of patients (12, 14, 15). Patient 1 had empyema and pulmonary abscess. However, Patient 2 presented with more frequent and severe infections and complications.

### 3.5. Pathologic Dentition and Bone Anomalies

They were found in 65–80% of patients (12, 14, 15), but some publications reported lower frequency −27% (11). We observed dental problems in both children, but there were neither abnormalities in bone density nor pathological fractures, scoliosis, or hyperextensible joints.

### 3.6. Facial Dysmorphism

Facial anomalies were visible only in Patient 1. The symptom is important for the diagnosis of the disease and occurs in over 90% of patients (11, 12, 14, 15). Usually, at an earlier age, the dysmorphic manifestations could be quite discrete and become more obvious until puberty. Therefore, in Patient 2, the presence of facial dysmorphism will be evaluated over time.

### 3.7. STAT3 Mutations

Prior to the detection of *STAT3* mutations, the diagnosis of HIES was made based on the scoring system [18]. The establishment of a *STAT3* pathogenic variant confirms the diagnosis. The *STAT3* gene plays an important role in the signal transduction of multiple pro- and anti-inflammatory cytokines [30, 31] and in the differentiation of Th17 cells, respectively, in IL-17 secretion [32]. The variant R382W in Patient 2 (DNA-binding domain) is one of the most common in AD-HIES [12], whereas c.1850 G > A (p.Gly617Glu) mutation (BC6 position of SH2 domain) in Patient 1 has been described only in a 19-year-old man by Schimke et al. [14] in 2010 and “classified as probably damaging.” According to the authors, the G617E variant arose de novo, and the patient presented with a high serum IgE level (>5,000 IU/mL), eczema, scoliosis, skin abscesses, and characteristic facies, without any pulmonary infections, pathologic fractures, or retained primary teeth. Although there is currently no reliable evidence that different mutations correlate with a specific clinical manifestation of the disease [12], patients with SH2 mutations have been reported to have a slightly higher arched palate, widened interalar distance, upper respiratory tract infections, and scoliosis, and those with DNA-binding domain mutations have a higher mortality rate [33]. Both our patients suffer from multiple infections, but in Patient 2, they were more severe. Patient 1 had a typical face dysmorphism.

### 3.8. Functional Studies on STAT3 Phosphorylation

The study of the intracellular STAT3 signaling activation pathway of T cells was performed with the BD Phosflow T-cell activation kit. The expression of phosphorylated STAT3 proteins was determined by flow cytometry. CD4+ and CD8+ cells from both patients were stimulated with IL-6 (100 ng/ml) and labeled with appropriate monoclonal antibodies for surface markers and intracellular phosphorylated proteins. Initially, labeled and unlabeled control beads were used to adjust the fluorescent compensations. Lyophilized control cells were tested as negative and positive controls. Subsequently, patient's samples and samples from corresponding age-matched healthy controls were tested simultaneously. The analysis was performed on FACS Canto II, FACS Diva software. The expression of intracellular phosphorylated proteins resulting from signaling pathway activation was determined by histogram based on the signal from Alexa Fluor 647 antiphosphoprotein antibody. We have determined the geometric mean fluorescence intensity (Geo MFI) value of each signal pathway of unstimulated and stimulated CD4+ and CD8+ T cells. The calculated ratio of Geo MFI stimulated to Geo MFI of unstimulated cells in patient-control pairs was used to estimate the deviation in the STAT3 signaling ability. The results showed that phosphorylation capacity via STAT3 in both patents was lower in comparison to healthy individuals for both CD4+and CD8+ cells (Table 2).

### 3.9. Immune Cells Subsets

STAT3 plays an important role in the regulation of B cells, CD4+, and CD8+ T cells. The differentiation of CD3+CD4+ cells is determined by the activation of the STAT3 pathway and related cytokines. However, the majority of AD-HIES patients did not show significant changes in these cell populations [15, 34]. Patients with AD-HIES were reported to have a decrease of CD4+ T-effector memory cells (TEMs) and an increase of CD4+ T-effector memory RA cells (TEMRAs) [35]. The percentage of T-lymphocytes in both of our patients was within reference values (Table 3). In Patient 1, the percentage of naïve CD4+ T cells (CD45RA+62L+) was significantly reduced, and the effector memory and effector subsets predominated (24.8 and 44.0% of CD4+ T cells, respectively). Siegel et al. [36] showed that STAT3 deficiency leads to a reduction of memory CD8+ T cells, which according to Ives et al. [37], is due to mutations in *STAT3* and *IL-21R* genes. Both of our patients had a decreased percentage of CD8+ T cells with a nearly normal distribution of naïve and memory cells (Table 3). In most studies, memory B cells in AD-HIES patients are reduced [15, 38, 39], and there are no correlations between low memory B cells, the ability for production of antibodies, and accompanied infections [38]. In our study, B cells were within the normal range. A slightly reduced percentage of NK cells was observed in Patient 2 (Table 3). The effects of STAT3 deficiency on NK cells need furder evaluation. The main change in T-lymphocytes associated with dominant negative *STAT3* mutations is a low percentage of Th17-cells [14, 15, 30, 40–42]. This population was extremely reduced in our patients as well (Table 3). Several authors [31, 41, 42] showed a great impairment in the ability of Th17 generation *in vivo* and *in vitro* to secrete IL-17 and 22 and generation of antigen-specific Th17 to different pathogens. T-cell function assessed by expression of the CD69 marker upon stimulation with phytohaemagglutinin in Patient 1 was retained but remarkably decreased via CD3 receptor pathway in comparison to age-matched healthy controls (2.1% of nonactivated PBMC expressed CD69+ and only 23.6% after T-cell receptor stimulation). At the time of the investigation, Patient 1 was not under corticosteroid or other immunosuppressive therapy.

### 3.10. Humoral Immunity and Vaccination-Induced Response

ANA, IgG, IgA, IgM, and IgG subclasses were normal in both cases. IgE was very high, especially in Patient 2 (Table 3), and a decrease in C4 was also observed. The ASO titer was high in Patient 1 but displayed optimal therapeutic response (results are not shown). Chandesriset et al. [15] described the following changes in serum immunoglobulin levels in AD-HIES patients: high serum IgG in 27%, high serum IgA–in 18%, high serum IgM–in 31%, and high serum IgE–in 96%. Moreover, low serum IgG was detected in 2% of the AD-HIES patients, low IgG1, IgG2, and IgG3–in overall 14%, and low serum IgA–in 13%. According to our data, Patient 1 had a protective level of Pneumococcal Capsular Polysaccharide (PCP) IgG and PCP IgG2, but the level of PCP IgA was decreased. The second patient had decreased protective levels of PCP IgG and PCP IgG2, but PCP IgA was normal. The titers of Haemophilus influenzae type B IgG and Tetanus toxoid IgG in both patients were comparable to those of the majority of children at that age, but the protective titer of antibodies against diphtheria toxoid in both cases was very low (Table 3). According to the literature, 21% of AD-HIES patients in a French study [15] have low antibodies against protein antigens (tetanus, diphtheria, or polio), 7%-low antibodies against *S. pneumoniae*, but 100%-normal antibodies against Haemophilus influence type B.

### 3.11. Cytokines

STAT3 is the basis of signal transduction of multiple cytokines and growth factors. [43]. On the other hand, STAT3 is involved in the differentiation of Th17 and the production of IL-17. A number of authors reported that in HIES patients, Th17 cells are significantly reduced and IL-17 production is severely impaired [40–42]. IL-17 is known to stimulate neutrophil proliferation and the production of colony-stimulating factor (G-CSF) and epithelial cell IL-8 [31, 44]. An impaired neutrophilic function is one of the main causes of poor response to pathogens, such as *streptococci* and *Candida* in patients with HIES. In AD-HIES, an imbalance between Th1 and Th2 responses, decreased production/expression of IFN-*γ* and relatively increased level/expression of IL-4, defects in IFN-*γ* and IL-12 signaling pathways, insufficient expression of some chemokines and adhesion molecules have been described and decreased expression of TGF-*β* and IFN-*γ* mRNA in circulating activated T-cells [45, 46]. The cytokine production capacity of HIES patients was tested in whole-blood cultures stimulated with heat-killed *Staphylococcus aureus*, *Candida albicans*, or a combination of IL-12/IL-18 [47]. The results revealed that IFN-*γ* production, in addition to IFN-*γ*/IL-10 ratio, was 10-30-fold lower in the HIES patients compared to the healthy subjects. In contrast, TNF, IL-1*β,* and IL-8 secretions were normal. The authors concluded that there was an imbalance towards a Th2 phenotype in HIES patients, which possibly contributes to the specific pattern of infections related to this particular PID. Holland et al. [6] demonstrated that the levels of TNF-*α*, IL-12p70, and IFN-*γ* produced by PBMC of patients with HIES were elevated in comparison to controls, depending on the stimulus. In our study, we tested the serum levels of 11 proinflammatory and anti-inflammatory cytokines in both patients (Table 4). The serum levels of proinflammatory cytokines (IFN-*γ*, IL-12p70, IL-1*β*, TNF-*α*, IL-18, and IL-6) and IL-2, IL-4, IL-5, GM-CSF in Patient 2 were increased, resembling a “cytokine storm.” After 4 years of symptomatic treatment, the follow-up values of the same cytokines were normal except IL-12p70, IL-1*β*, IL-6, and IL-18. However, the values were reduced several-fold compared to the previous testing. In the fifth year after the treatment initiation, the levels of IL-2, IL-4, TNF-*α*, GM-CSF, and IL-18 were elevated again, reflecting some subclinical manifestations and exacerbation of persistent dermatitis. The elevated proinflammatory cytokines TNF-*α* and IL-6 after 6 years of treatment probably preceded the reported episode of pulmonary complication several months later. In Patient 1, the Th1 profile and a relatively elevated level of IL-4 and GM-CSF predominated at the time of diagnosis. After the initiation of anti-infectious prophylaxis (no severe infections so far), the values of the cytokines became normal except IL-18, which had a sevenfold decrease. In our opinion, the observed changes in cytokines in both patients were mostly associated with concomitant infections. In a patient with the common *STAT3* variant who presented with a more severe course, the cytokine disturbance was most significant and persistent over time. The changes in cytokine levels could serve as an important laboratory indicator of therapeutic response to infections and an early marker of recurrence of complications. More studies with a larger number of patients are needed to confirm or reject these considerations.

## 4. Concluding Remarks

Several hundred cases of AD-HIES have been described in the medical literature so far, and few studies have focused on the genotype-phenotype correlations and the changes in humoral and cellular immune response and cytokine profiles. The study participants fulfill the criteria for AD-HIES with an approximately equal score index. However, they presented with different immunological findings and symptom severity, probably due to the functional impact of the individual *STAT3* variants. We believe that our comparative approach, based on detailed clinical and laboratory information, will contribute to the enrichment of data for this rare syndrome. In this context, it is important to continue efforts to establish immunological biomarkers that might be predictive or supportive for patient evaluation and management.

## Figures and Tables

**Table 1:** Hyper IgE Syndrome Scores and *STAT3* mutation data.

Symptoms	Patient 1	Points	Patient 2	Points
Highest IgE	2180 IU/ml	10	9740 IU/ml	10
Skin abscesses	3-4	4	3-4	4
Pneumonia	2	4	2	4
Parenchymal lung abnormalities	Pneumatocele	8	Pneumatocele	8
Other serious infection	Empyema and abscesses pulmonum	4	Abscesses retroperitonealis and subfrenicus dextra	4
Fatal infection	Absent	0	Peritonitis, perforation colon transversum	4
Highest eosinophils (10^9^ L)	0.94	6	0, 7	6
Newborn rash	Absent	0	Absent	0
Eczema (worst stage)	Moderate	2	Moderate	2
Sinusitis/otitis	1–3	1	Absent	0
Candidiasis	Finger/nail	2	Systemic	4
Retained primary teeth	>3	8	>3	8
Scoliosis, max curve	Absent	0	Absent	0
Fractures with little trauma	Absent	0	Absent	0
Hyperextensibility	Absent	0	Absent	0
Characteristic face	Mild	2	Absent	0
Increased nose width (interallar distance)	1-2 SD	1	Absent	0
High palate	Present	2	Present	2
Midline anomaly	Absent	0	Absent	0
Lymphoma	Absent	0	Absent	0
Young age add-on	<1 year	7	<1 year	7
SCORE		61		63
*STAT3* mutations	NM_139276.2(*STAT3*):с.1850 G > A(p.Gly617Glu)		NM_139276.2 (STAT3):c.1145 G > T (p.Arg382Leu)	

**Table 2:** Evaluation of STAT3 signaling pathway activation in patients.

Individuals tested	STAT3 activation in CD4+		STAT3 activation in CD8+	
Patient 1	CD4+ (U) Geo MFI	5	CD8+ (U) Geo MFI	5
	CD4+ (S) Geo MFI	16	CD8+ (S) Geo MFI	17
	**Geo MFI index**	**3.2**	**Geo MFI index**	**3.4**

Healthy control 1	CD4+ (U) Geo MFI	7	CD8+ (U) Geo MFI	5
	CD4+ (S) Geo MFI	42	CD8+ (S) Geo MFI	40
	**Geo MFI index**	**6**	**Geo MFI index**	**8**

Patient 2	CD4+ (U) Geo MFI	13	CD8+ (U) Geo MFI	9
	CD4+ (S) Geo MFI	32	CD8+ (S) Geo MFI	37
	**Geo MFI index**	**2.5**	**Geo MFI index**	**4.1**

Healthy control 2	CD4+ (U) Geo MFI	139	CD8+ (U) Geo MFI	170
	CD4+ (S) Geo MFI	901	CD8+ (S) Geo MFI	971
	**Geo MFI index**	**6.5**	**Geo MFI index**	**5.7**

U: unstimulated; S: stimulated; Geo MFI: geometric mean fluorescence intensity; Geo MFI index: ratio of Geo MFI of stimulated to Geo MFI of unstimulated cells.

**Table 3:** Immunological phenotype of Patients 1 and 2.

Immune phenotype/marker (units)	Patient 1	Patient 2	Reference range
WBC (cells × 10^9^/L)	7.3	7.0	4.5 ÷ 13
ANC (cells × 109/L)	1.97	2.0	1.8 ÷ 8.0
ALC (cells × 109/*µ*L)	3.79	3.8	1.5 ÷ 6.5
Eos (%)	12	10.2	0.0 ÷ 6.0
CD3+ (%Ly)	63	77	66 ÷ 76
CD3+DR+ (%Ly)	9	5^*∗*^	9.5 ÷ 17
CD3+CD4+ (%Ly)	36	45^*∗∗*^	33 ÷ 41
CD45RA+62L+ from CD4+ (%Ly)	24.8^*∗*^	73.6	46 ÷ 99
CD45RA-62L+ from CD4+ (%Ly)	6.4	19.8	0.35 ÷ 100
CD45RA-62L− from CD4+ (%Ly)	24.8^*∗∗*^	4.8	0.27 ÷ 18
CD45RA+62L− from CD4+ (%Ly)	44.0^*∗∗*^	1.8	<1.8
CD3+CD8+ (%Ly)	19^*∗*^	21^*∗*^	27 ÷ 35
CD45RA+62L+ from CD8+ (%Ly)	45.5	68.1	16 ÷ 100
CD45RA-62L+ from CD8+ (%Ly)	5.6	5.9	1 ÷ 6
CD45RA-62L- from CD8+ (%Ly)	23.6	12.4	5 ÷ 100
CD45RA+62L- from CD8+ (%Ly)	25.3	13.6	15 ÷ 41
CD19+ (%Ly)	18	20	12 ÷ 22
CD3-CD16 + 56 (%Ly)	14	6^*∗*^	9 ± 16
CD3+CD16 + 56+ (%Ly)	10	3	4 ÷ 26
CD25+CD127low (%Ly)	8.4	6.2	5 ÷ 10
CD4+CD161+CD196+ (%Ly)	2.34; 2.0^*∗*^	1.2^*∗*^	12.5 ÷ 14.9

IgG (g/l)	16.079	10.64	5.40–16.10
IgG1(g/l)	10.902	8.482	4.23–10.6
IgG2 (g/l)	2.5	2.738	0.72–4.3
IgG3 (g/l)	0.681	0.934	0.127–1.731
IgG4 (g/l)	0.428	0.24	0.016–1.151
IgA (g/l)	1,742	0.75	0.50–2.80
IgM (g/l)	1.400	2.41^*∗∗*^	0.5–1.90
IgE (U/ml)^*∗∗∗*^	2180; 1995; 1104; 1414; 2000^*∗∗*^	431; >2500; 9740; 22400^*∗∗*^	<87
Aspergillus fumigates-specific IgE	Negative	Positive	Negative
C3 (g/l)	1,557	1.303	0.75–1.65
C4 (g/l)	0.510	0.118^*∗*^	0.20–0.65
ANA (U/ml)	1 : 160	1 : 160	1 : 160
ASO (U/ml)	1066; 522 U/ml^*∗∗*^	12	<200

PCP IgG (mg/L)	85.6	15.4^*∗*^	>30
PCP IgG2 (mg/L)	32.0	2.88^*∗*^	>11
PCP IgA (mg/L)	1.03^*∗*^	74.6	NA
PCP IgM (mg/L)	3.53^*∗*^	209.4	NA
Hib IgG (mg/L)	16.5	6.43	>0.15
DT IgG (mg/L)	0.04^*∗*^	0.025^*∗*^	>0.1
TT IgG (mg/L)	0.11	0.35	>0.1

^*∗*^Low values; ^*∗∗*^high values; ^*∗∗∗*^measurement of IgE is in flux with approximately one-year follow-up intervals. WBC: white blood cell count; ANC: absolute neutrophil count; ALC: absolute lymphocyte count; Eos: eosinophils; PCP: pneumococcal capsular polysaccharide; Hib: *Haemophilus influenzae type B*; DT: diphtheria toxoid; Td: tetanus toxoid; NA: not applicable. The estimation of PCP IgA and IgM values was based on the comparison with the titer of the same antibodies in children tested in our laboratory (data not published).

**Table 4:** Serum levels of cytokines investigated in Patients 1 and 2.

Cytokine	Concentration (pg/ml)						
	Patient 1		Patient 2				Reference range^*∗*^
	At the time of diagnosis, without prophylactic treatment	After 2 y of prophylactic treatment	At the time of diagnosis, without prophylactic treatment	After 4 y of prophylactic treatment	After 5 y of prophylactic treatment	After 6 y of prophylactic treatment	
IFN-gamma	143.85	14.5	244.37	6.47	17.75	3.92	8.08 ± 25.32
IL-12p70	54.46	0.0	50.96	2.83	2.63	0.0	0.90 ± 1.26
IL-13	1.17	0.0	3.81	0.0	0.70	0.56	0.09 ± 0.26
IL1beta	1.76	0.0	47.04	1.32	1.76	0.0	0.09 ± 0.23
IL-2	0.0	0.0	404.17	0.87	7.93	0.0	0.57 ± 1.41
IL-4	8.42	0.0	217.52	0.0	6.59	0.0	0.0
IL-5	1.22	0.0	10.39	0.0	0.61	0.0	0.94 ± 1.26
IL-6	25.71	0.0	18.46	1.70	1.70	3.12	0.07 ± 0.24
TNF-alpha	2.09	0.0	9.64	0.0	6.26	4.17	0.08 ± 0.28
GM-CSF	5.11	0.0	31.46	0.0	23.39	0.0	0.64 ± 1.34
IL-18	113.32	16.0	43.61	22.4	43.52	4.53	1.67 ± 1.75

^*∗*^The values are laboratory specific based on healthy controls tested.

## Data Availability

The data used to support the study are included within the manuscript.
